# An Association of Mucinous Adenocarcinoma With Chronic Peri-Anal Fistula: A Brief Review of Pathophysiology of Rare Tumor

**DOI:** 10.7759/cureus.8882

**Published:** 2020-06-28

**Authors:** Muhammad Tahir, Jawaria Rahman, Tayyaba Zubair, Abdul Basit

**Affiliations:** 1 Pathology, Case Western Reserve University School of Medicine, Cleveland, USA; 2 Pathology, City of Hope, Comprehensive Cancer Center, Monrovia, USA; 3 Internal Medicine, Coney Island Hospital, Brooklyn, USA

**Keywords:** chronic peri-anal fistula, anal carcinoma, mucinous carcinoma, peri-anal mucinous adenocarcinoma, mucinous adenocarcinoma

## Abstract

An anal fistula is a condition that has been discussed by various authors without coming to a consensus. The fistula of the anus is a passage that leads from the rectum to the anus. A peri-anal adenocarcinoma is an abnormal growth of cells in the anal rectal area. Establishing the relationship between the two, a lot of information was obtained from the existing literature which has used to come up with solutions to the objective of the study. The review was conducted systematically and included observational retrospective, case studies, and case series to demonstrate the data of valuable research. The articles were searched in PubMed, MEDLINE, and Google scholar using the keywords “chronic perianal fistula”, “anal carcinoma”, “mucinous adenocarcinoma”, and “perianal mucinous adenocarcinoma”. Among 50 journal articles, we chose 33 studies describing the clinical sign and symptoms, pathophysiology, etiology, and association between mucinous anal adenocarcinoma and chronic peri-anal fistula. After quality assessment, eight case studies and series were selected, in which seven of them showed the origin of mucinous anal adenocarcinoma from chronic peri-anal fistula. The existence of a long history of fistula-in-ano and the exclusion of any additional carcinoma in the body necessitates the analysis of mucinous adenocarcinoma arising from benign fistula. Likewise, the presence of fistula ought to typically precede that of carcinoma by 10 years, and this is one of the criteria for diagnosis. However, more investigation should be conducted to gain full information about the connection between these two entities.

## Introduction and background

Peri-anal fistulas can be found frequently in the clinical setting, but the malignant transformation of these fistulas is not common. The development of anal mucinous adenocarcinoma (MA) from chronic peri-anal fistula is rare, and only a few such cases have been reported in the past [1]. Anal fistulas usually develop from an anal abscess, and the background of the fistula is regularly very typical. The patient with anal fistulas, along with abscess, generally has signs and symptoms of peri-anal swelling, pain with defecation, fever, lethargy, purulent discharge, and rectal discomfort [2]. Overall, 37% of patients develop a fistula from an anal abscess. The occurrence of anal fistula is higher in females than in males with 50% and 31%, respectively [3].

Cancer of the peri-anal fistula is an erratic disease. It is predominantly associated with chronic anal fistula. The MA accounts for 2-3% of all anorectal malignancies. In most cases, prolonged duration inflammation is the possible reason for fistula-related carcinomas [4]. Diagnosis is frequently not made on time because the disease appears very slowly with fewer symptoms. The neoplasm tends to develop into large masses before they can be identified [5].

MA is a diverse clinical and pathological entity within the range of colorectal neoplasms and reports nearly 10-15 % of cases. Causes related to MA growth and progress have not been entirely known, but clinical observational evidence can guide us to the best understanding of its etiology [6]. To satisfy the definition of MA as highlighted by the World Health Organization, more than 50% of the tumor should comprise extracellular mucin [7]. Chronic inflammation presumably plays a significant role in carcinogenesis. Colorectal carcinomas (CRCs) occur primarily in areas affected by inflammation, but the mechanism of carcinogenesis is not yet totally understood [8-10]. As indicated by some studies, the peri-anal MA is linked to chronic anal fistula. Howbeit, that is not always the case; very limited research has been conducted to investigate the connection between peri-anal fistula and MA. Plenty of other studies will need to be performed to have a complete picture of this relationship.

There are worldwide differences in the incidence of mucinous carcinomas (MCs), with low rates in Asian countries and increased rates in the western world. Furthermore, MC is more often diagnosed in patients suffering from inflammatory bowel diseases or Lynch syndrome, and a high rate has been seen in patients with radiotherapy-induced CRCs. These outcomes are indicative of various oncogenic origin [6]. The purpose of this review is to perform an analysis of the emergence of MA from chronic anal fistula and understand the pathophysiology of this rare malignancy.

## Review

Method

We performed this study systematically using the Preferred Reporting Items for Systematic Reviews and Meta-Analyses (PRISMA) [11]. To search for the information, we used the various resources but mainly selected PubMed for gathering the majority of the data. Resources such as MEDLINE, WHO website, PubMed Central, WebMD, and Google Scholar were also searched. The keywords used for searching included chronic peri-anal fistula, anal carcinoma, MC, adenocarcinoma (AD), and peri-anal MA. We concentrated on the adult population from all over the globe without prejudice between gender, race, nationality, and ethnicity. Nevertheless, several publications demonstrating a correlation among various age groups have also been added. Among all relevant articles, a quality assessment checked was performed using A MeaSurement Tool to Assess Systematic Reviews (AMSTAR) checklist, and multiple publications were omitted [12]. All data was collected scientifically and ethically.

Result

We retrieved 2,119 studies from PubMed and 16,900 from Google scholar studies using the search term “chronic peri-anal fistula” and “mucinous anal adenocarcinoma”. The combined use of the search term “relationship between chronic peri-anal fistula and mucinous anal adenocarcinoma” provided us with 13,303 PubMed and 18,500 Google Scholar research papers. A total of 50 articles related to our subject were picked from these findings. After implementing the exclusion/inclusion criteria and removing the repetition, we ultimately got 33 research papers that were considered for review. The majority of the related articles that were selected were case studies and series, which discussed the clinical sign and symptoms, pathophysiology and etiology, and the relationship between chronic peri-anal fistula and mucinous anal AD. After careful consideration, eight case studies and series were chosen, in which seven of them demonstrated the association between chronic peri-anal fistula and mucinous anal AD.

Discussion

**Figure 1:**
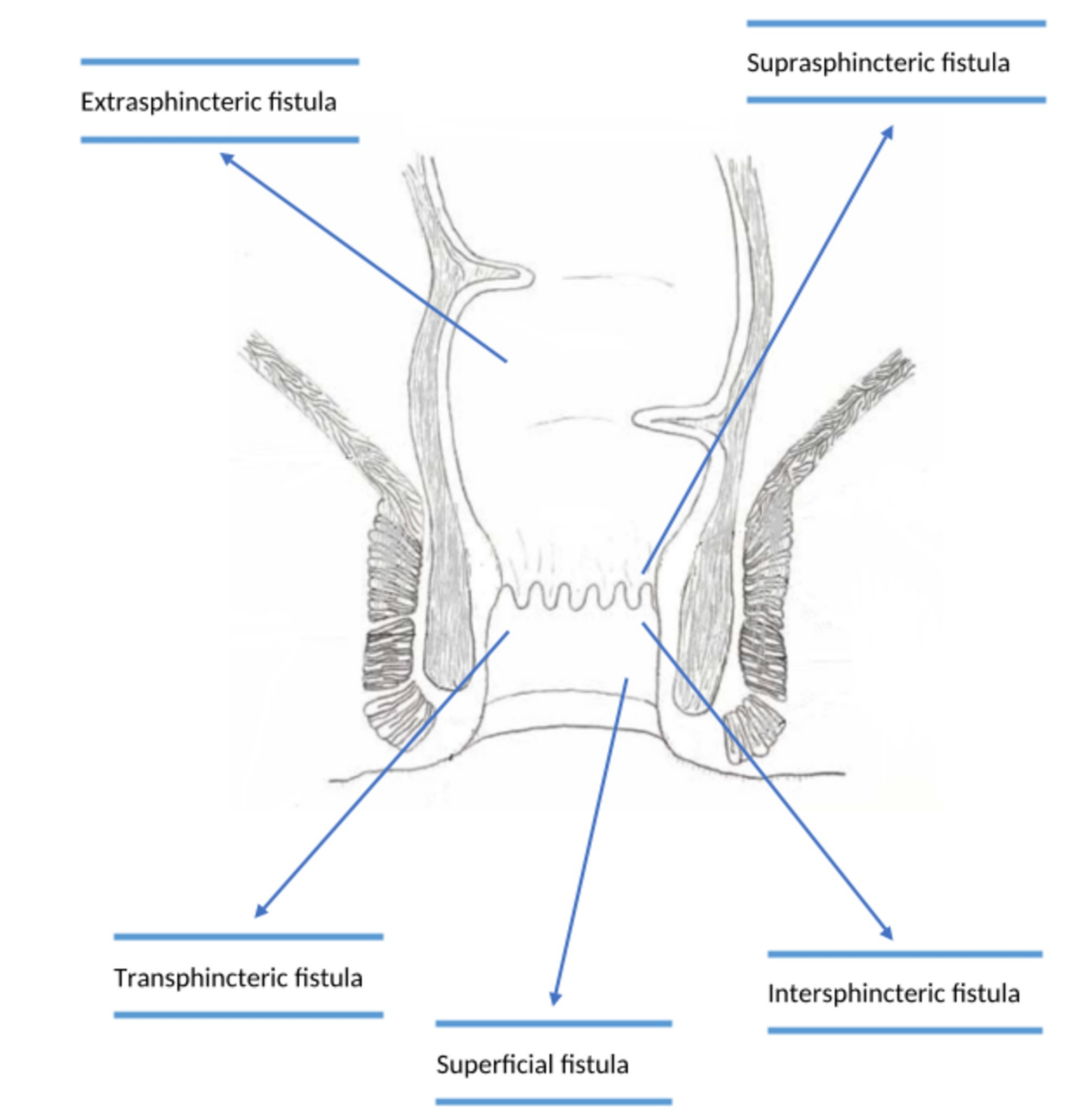
Classification of peri-anal fistula

An anal fistula (often called fistula-in-ano) is most of the time a consequence of a past or current anal abscess. This occurs in up to half of patients with abscesses. Typical anatomy consists of small glands directly inside the anus. The fistula is the channel that builds beneath the skin and links the blocked infected glands to an abscess. A fistula can exist without an abscess and may connect to the skin of the buttocks close to the anal opening. Fistulas have categorized through their association with the parts of the anal sphincter complex (the muscle that controls stool to pass). They are as follows: superficial fistula, transsphincteric, extrasphincteric, suprasphincteric, and intersphincteric, as shown in Figure 1. The most common one is the intersphincteric, and the least common is extrasphincteric. These classifications are necessary for assisting the physicians in making their treatment choices [13].

The emergence of MA from peri-anal fistula is still perplexing. The area around anus consists of both squamous and columnar epithelium, and therefore carcinoma can be squamous cell carcinoma or AD [14]. The anal canal acts as an anatomical transition area between the peri-anal squamous mucosa and the columnar epithelium. The underlying pathophysiology that leads to malignant transformation is still unknown, but the chronic inflammation with frequent epithelial regeneration has considered instrumental [15].

**Figure 2:**
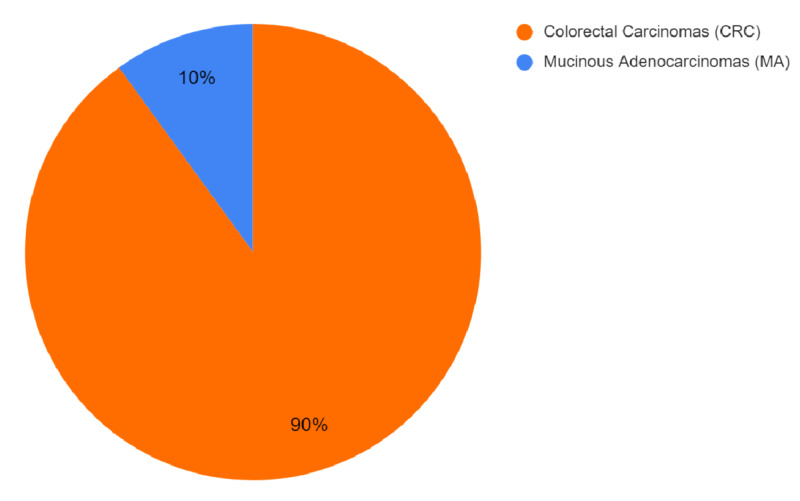
Ratio of mucinous adenocarcinoma relative to all colorectal cancers

**Figure 3:**
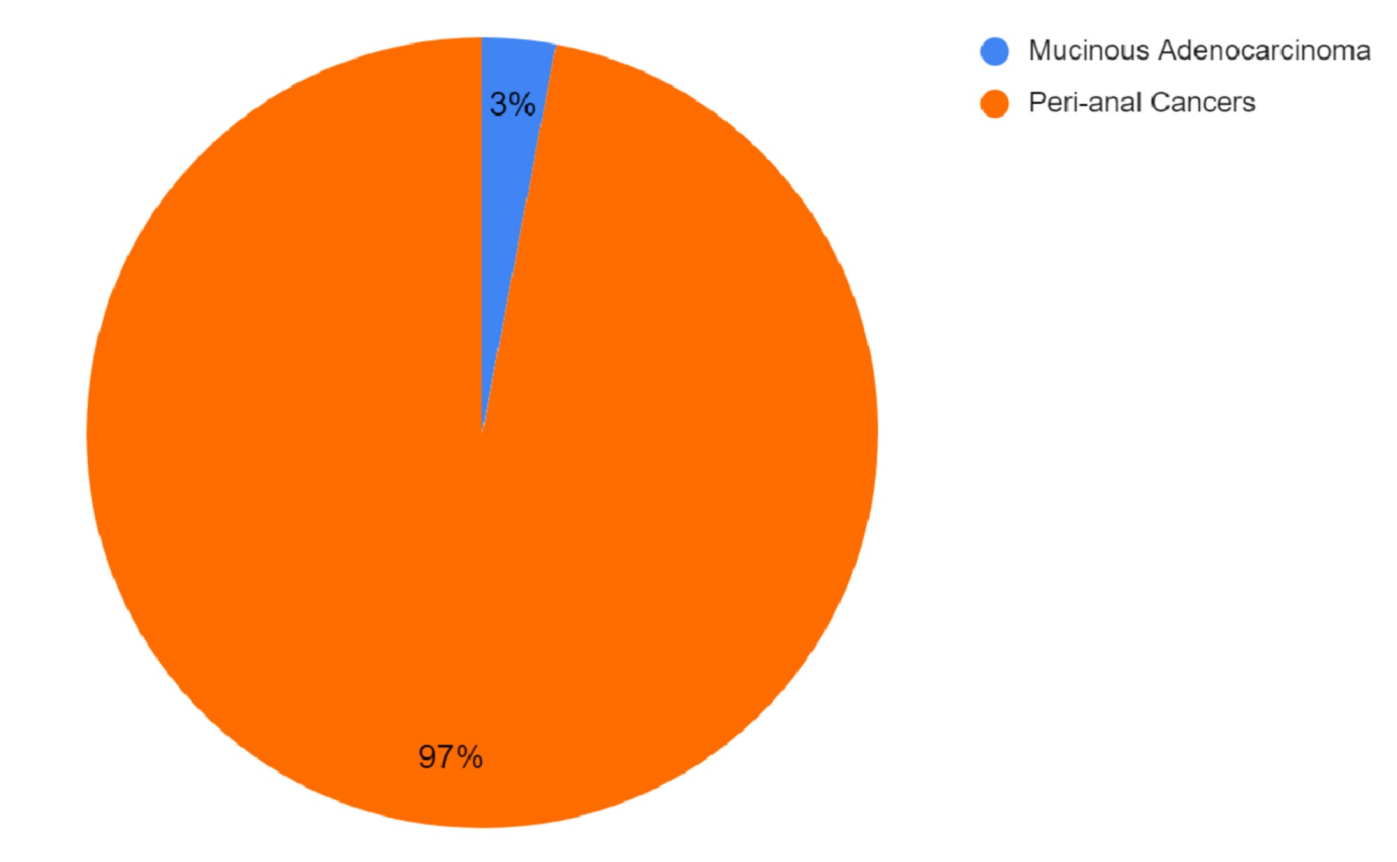
Ratio of mucinous adenocarcinoma relative to peri-anal carcinomas

MA is a subcategory of colorectal cancer, accounting for almost 10% of all colorectal cancer (CRC), as shown in Figure 2, and roughly 2-3% peri-anal cancers are MAs, as shown in Figure 3 [16,17].

This sort of cancer comprises malignant cells that form an enormous quantity of extracellular mucin, and the histopathological identification needs to have more than 50% mucinous element of the tumor mass [7]. The prognostic findings of MA patients stayed questionable. Few investigators have put forward that patients with colorectal MA have a poorer prognosis than those with AD, though others have indicated no discrepancy [17-22].

The most common causes of MC are inflammatory bowel disease and Lynch syndrome, and a higher rate is seen in patients with radiotherapy-induced CRCs [6]. A handful of cases have indicated the origin of MA from chronic peri-anal fistula.

Further details revealed the different techniques to diagnose the MA, which includes endoanal ultrasonography (EAUS), which is the most efficient one and shows the specific attributes of MA arising from chronic peri-anal fistula. Sonography-guided biopsy under anesthesia is quite beneficial for the definitive diagnosis of the tumor. In this manner, regular evaluation with EAUS ought to be suggested for patients with long-standing peri-anal fistula, particularly those with dynamic clinical manifestations. Once the malignancy is suspected, vigorous sonography-guided biopsy under anesthesia should be carried out, which may empower the early findings, treatment, and favorable long-term benefits [23].

MCs of the colon and rectum grow and metastasize faster than non-MCs. It is believed that the pathological and clinical aspects resulted in decrease curability, consequent relapse, and an adverse prognosis. For that reason, surgeons recognized that more proactive surgical treatment, including a wide range of lymph node dissection and resection of adjacent organs that are probably involved, must be delivered from time to time to better the postoperative prognosis of the patients with colorectal MC [24].

The pathogenesis has not yet been entirely explained. Traube et al. described the theory of constant mucosal regeneration within the fistula, which can lead to dysplastic changes that may result in developing AD [25]. Symonds et al. suggested that the neoplasm categorized as MC is made up of glands secreting pools of mucus deep inside the infiltrating part of the tumor [26]. Pihl et al. stressed that MC should be limited to neoplasms in which mucin prevails [27]. Moreover, Wu et al. and Connelly et al. mentioned that a tumor in which the mucinous portion is at least 60% of the neoplasm should be considered as an MC [28,29]. The biologic importance of MC of the colon and rectum continued to be contentious [30,31]. Overall, the prognosis of the patients diagnosed with colorectal MC is worse [23].

**Figure 4:**
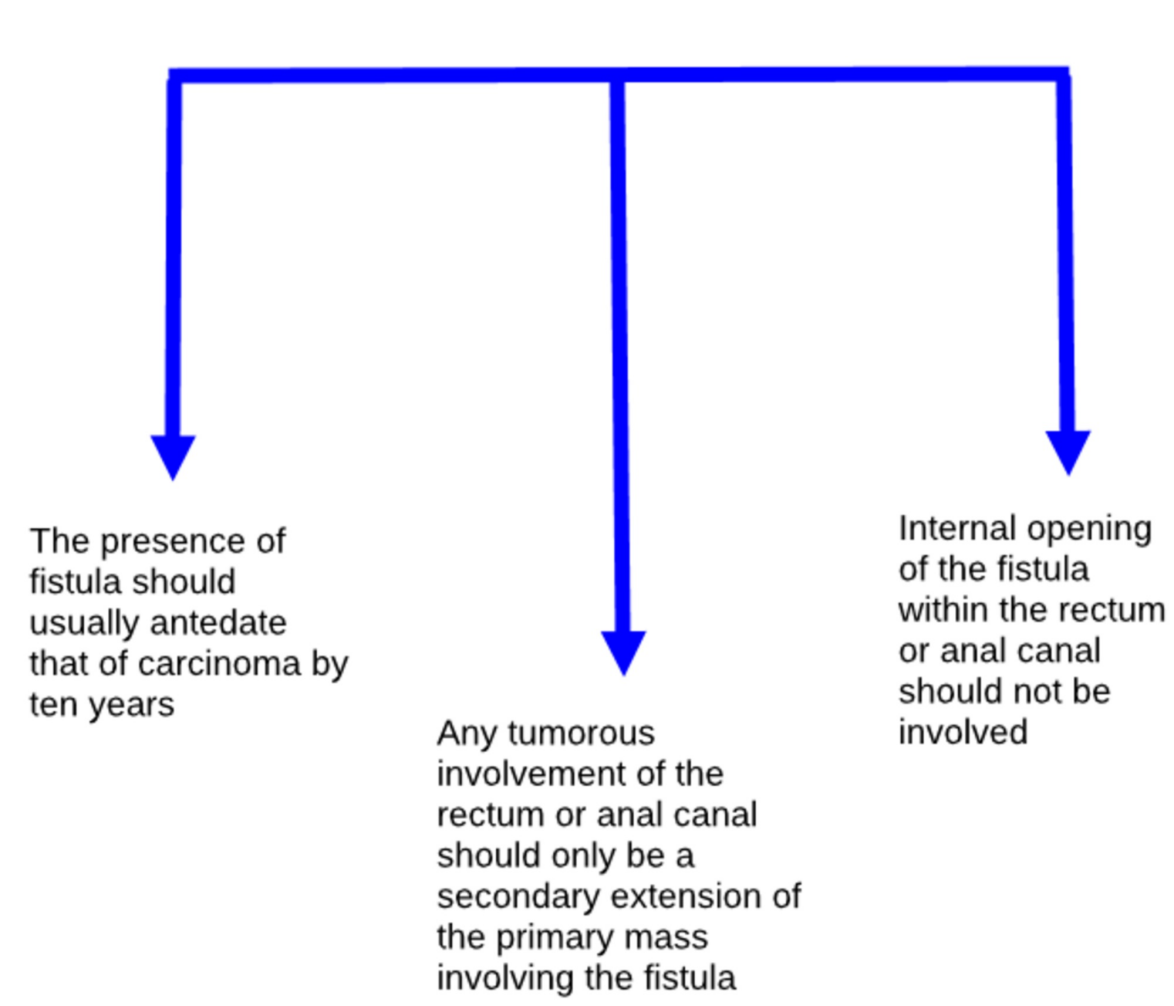
Three-point criteria presented by Prasad et al. emphasizing an association of mucinous adenocarcinoma with chronic peri-anal fistula [1]

However, many studies have been conducted to clarify the pathogenesis of MA and its origin from chronic peri-anal fistula. Yet, we still have a gap in establishing the precise diagnosis and need more clinical-based evidence to prove the association between two. A study conducted by Santos et al. conducted a study on patients with constant peri-anal fistulas who gradually grew two peri-anal ulcerated sores close to the outer orifices of the fistulas, ultimately reaching out as a pararectal tumor. The histopathological biopsy confirmed the diagnosis of MA [32]. Another study was conducted by Haliloğlu et al. to study the results of magnetic resonance imaging (MRI) of MA arising from peri-anal fistula, and based on the study, MRI is the choice of imaging technique for the diagnosis of MA arising from peri-anal fistula. The study also reported that a fistula tract biopsy of the two patients with a history of chronic peri-anal fistula showed MA [15]. In another study, Prasad et al. reported the origin of MA in a patient with chronic anal fistula, which was confirmed by transrectal ultrasound-guided biopsy and consecutive histopathological examinations. The study summed up three criteria needed for the diagnosis of MA: (1) the presence of fistula ought to usually precede that of carcinoma by 10 years, (2) any tumorous association of the rectum or anal canal should only be a secondary extension of the primary mass involving the fistula, and (3) internal opening of the fistula within the rectum or anal canal should not be included, as shown in Figure 4 [1].

Toyonaga et al. showed the significance of EAUS of MA arising from chronic peri-anal fistula. The study demonstrated a typical pathology of MA with multiloculated complex echoic mass along isoechoic solid components communicating with a trans-sphincteric fistula, as shown by a sonography-guided biopsy under anesthesia [23]. Another study was conducted by Papaconstantinou et al. which reported the root of the MA within a chronic peri-anal fistula in Crohn’s disease. The study proved that the constant mucosal regeneration inside a fistula in Crohn’s disease tends to be a dominant pathogenetic mechanism. The mucosal biopsies collected from the lesions and the underlying fistulous tracts showed granulomatous tissue infiltrated by mucus-producing AD [33]. The study by Díaz-Vico et al. involved three patients and demonstrated that all three patients with chronic peri-anal fistula had developed MA over time. In the study, they emphasized that histopathological diagnosis remains the gold standard method of diagnosis. The presence of extracellular mucinous lakes encompassed by well-differentiated dilated tortuous glands, nerves, and vessels confirmed the diagnosis of MA [34]. One more study conducted by Bo-Lin Yang et al. based on retrospective case reviews identified three patients with chronic peri-anal fistula who were subsequently found to developed peri-anal MA on biopsy [35]. Table 1 summarizes all the aforementioned studies.

**Table 1:** An association of mucinous adenocarcinoma with chronic peri-anal fistula in relevant studies IBD, inflammatory bowel disease; TNF, tumor necrosis factor

No.	Author	Year	Type of Study	Purpose of the Study	Results/Conclusion
1	Santos et al. [32]	2014	Case report	To confirm the origin of mucinous adenocarcinoma from chronic peri-anal fistula	This study showed a patient with constant peri-anal fistulas who gradually grew two peri-anal ulcerated sores close to the outer orifices of the fistulas, which ultimately reached out as a para-rectal tumor. Later, the histopathological biopsy confirmed mucinous adenocarcinoma. The occurrence of a long history of fistula-in-ano and the exclusion of another carcinoma in the body permit the diagnosis of carcinoma emerging from fistula.
2	Haliloğlu et al. [15]	2013	Case series	To study the results of the MRI of mucinous adenocarcinoma arising from peri-anal fistula	According to the study, MRI is the choice of imaging technique for the diagnosis of mucinous adenocarcinoma arising from peri-anal fistula. The study showed fistula tract biopsy of the two patients with a history of the chronic peri-anal fistula, which confirmed as a mucinous adenocarcinoma.
3	Hugen et al. [6]	2014	Review article	To illustrate the etiology of mucinous adenocarcinoma	This study explained the different causes of mucinous carcinoma such as genetic alterations, lifestyle variations, dietary changes, autoimmune diseases such as IBD, and radiation therapy, but does not explain that chronic anal fistula is one of the causes of anal mucinous adenocarcinoma.
4	Prasad et al. [1]	2018	Case report	To explain the origin of mucinous adenocarcinoma from chronic anal fistula	This study showed the arising of mucinous adenocarcinoma in a patient of chronic peri-anal fistula who has symptoms of pain and recurrent discharge, which was later confirmed by transrectal ultrasound-guided biopsy and consecutive histopathological examinations. The study summed up three criteria needed for the diagnosis of this condition: (1) the presence of fistula ought to usually precede that of carcinoma by 10 years, (2) any tumorous association of the rectum or anal canal should only be a secondary extension of the primary mass involving the fistula, and (3) internal opening of the fistula within the rectum or anal canal should not be affected. The study met the last two criteria; however, the period of the occurrence of the chronic peri-anal fistula was four years.
5	Toyonaga et al. [23]	2017	Case series	To know the significance of endo-anal ultrasonography of mucinous adenocarcinoma emerging from chronic peri-anal fistula.	This study revealed a typical pathology of mucinous adenocarcinoma with multiloculated complex echoic mass along isoechoic solid components communicating with a transsphincteric fistula as shown by a sonography-guided biopsy under anesthesia. This case series study involved three patients with a 5- to 20-year history of anal fistula.
6	Papaconstantinou et al. [33]	2015	Case report	To clarify the root of the mucinous adenocarcinoma within a chronic peri-anal fistula in Crohn’s disease	In this study, mucosal biopsies were collected from the lesions and the underlying fistulous tracts, which showed granulomatous tissue infiltrated by mucus-producing adenocarcinoma. The study proved that the constant mucosal regeneration inside a fistula in Crohn’s disease tends to be a common pathogenetic mechanism for developing carcinoma. At the same time, immunosuppressants and anti-TNF agents may also lead to the malignant transformation.
7	Díaz-Vico et al. [34]	2019	Case series	To study the relationship of mucinous adenocarcinoma with chronic peri-anal fistula.	The study involved three patients and demonstrated that all three patients aged between 62 and 77 years with chronic peri-anal fistula had developed mucinous adenocarcinoma over time. Histopathological diagnosis remains the gold standard method of diagnosis. The presence of extracellular mucinous lakes encompassed by well-differentiated dilated tortuous glands, nerves, and vessels confirmed the diagnosis.
8	Yang et al. [35]	2009	Retrospective observational study	To study the development of peri-anal mucinous adenocarcinoma from chronic per-anal fistula	The study was a retrospective chart review and identified three patients with chronic peri-anal fistula who were subsequently found to have developed peri-anal mucinous adenocarcinoma on biopsy. Two of three patients who underwent irradiation and chemotherapy were alive at 24 and 28 months of follow-up, respectively.

Based on the studies reviewed, MA arising from long-standing peri-anal fistulas is uncommon. However, the occurrence of a long history of fistula-in-ano and the exclusion of another carcinoma in the body allows the diagnosis of MA emerging in a benign fistula. Also, the presence of fistula should usually antedate that of carcinoma by 10 years, and this is one of the pieces of evidence found in studies. On gross analysis, the tumor appears tan whitish to pinkish with cut-surface, might be germinal scar or necrosis, usually well-defined, and could be capsulated, smooth, cystic, or solid with even distribution with or without modularity. Furthermore, chronic mucosal regeneration plays a vital role in the pathogenesis of MA. The confirmatory diagnosis of mucinous AD includes the histopathological findings of the presence of extracellular mucin encircled by dilated tortuous glands.

Limitations

The research has various limitations that could affect the accuracy of the results. Foremost, the number of people with the condition is very minimal, thus making it impossible to get enough data for analysis. Most of the investigation was based on a small sample size in their report. Another limitation could be that the research was conducted based only on the literature. Thus, the reliability of the information or data was dependent on already existing research studies. Another limitation is that the condition is very hard to diagnose until the symptoms usually manifest themselves during the last stages, and the duration of time to follow the results of an association between the two entities makes the researcher bore or quit from the study. Therefore, the delayed analysis could compromise the accuracy of the results.

## Conclusions

MA arising from chronic peri-anal fistula is an uncommon occurrence. Still, it is visible that the two conditions have an association with each other, where the chronic disease causes the other, though not all the times. Understanding this relationship may be challenging, especially the diagnosis, as the signs manifest themselves at the later stages. The condition can be controlled if it is diagnosed early enough. A high level of clinical suspicion is required for early diagnosis, which builds the extension for therapeutic medical and surgical procedures. Imaging is very helpful in identifying the growing malignancy and staging the disease, thereby directing early and effective treatment. The histopathological assessment is prudent to neglect the chance of missing an underlying malignancy. Establishing the relationship between the two conditions will assist in decreasing the tumor burden for the hospital, patient, and government beforehand, and will also help focus on the patients to improve their living standards and handle and endure their chronic illness in comparison with the suffering of complications of malignancy, which will deteriorate their life physically, mentally, and monetarily.
